# On Diphtheria: With Cases

**Published:** 1885-06

**Authors:** Alfred Sheen

**Affiliations:** Senior Surgeon, Cardiff Infirmary


### ON DIPHTHERIA: WITH CASES.

By ALFRED SHEEN, M.D., Senior Surgeon, Cardiff Infirmary.

DIPHTHERIA, like its ally, scarlet fever, is a treacherous disease which always rivets our attention when we have it to deal with, and is invariably a source of great anxiety to us in our efforts to promote recovery. Having, some time ago, had a series of cases in one family, with many points of interest arising out of them, perhaps a history of these cases, with some remarks bearing upon them and upon the disease in general, might be deemed worthy of consideration.

I will first give the cases as briefly as possible:

I. Mary R., æt. 5½. I was called to see this child on Monday, June 24th, at 9 a.m. It was lying on the sofa with its head thrown back, eyelids half-closed, mouth open. The breathing was roughened, and there was occasional cough and some restlessness. Offensive odour with the breath. What examination of the throat could be made revealed blue-greyish thickened patches on the tonsils and all parts of the back of the throat which could be seen. Child quite sensible. P. 148, T. 103·4°.

℞. Potass. Chlorat. ʒiij, Glycerin. ʒiij.

Aq. ad ℥vj, ℥ss. every 3 hours.

℞. Tr. Aconit. ♏xxx, Aq. ad ℥iv.

ʒi. every $\frac{1}{4}$-hour 4 times, then every $\frac{1}{2}$-hour.

2.30 p.m. P. 148, T. 102°. Child no better.

8 p.m. P. 160. Consultation suggested.

9.30 p.m. P. 160, T. 104·4°. Dr. Taylor saw the patient with me. He concurred in the treatment, and suggested the addition of a spray of sulpho-carbolate of soda, 4 grs. to the oz. The child gradually got worse, and died at 5 a.m. on the 25th, from asthenia, and not from suffocation.

This child, a particularly robust and healthy one, was taken ill on the previous Wednesday, but was out on Saturday, and apparently had no throat symptoms till Sunday night, 10 hours before I was called in, when raucous breathing and a cough of a peculiar character came on. The eldest child was just recovering from what was deemed a slight illness with sore throat, which was treated solely by domestic remedies.

The family consisted of a father and mother; children aged respectively about $9\frac{1}{2}$, 8, $5\frac{1}{2}$, and 3; housemaid, cook, and nurse; and only two of these escaped throat illnesses, namely, the mistress and the nurse.

On the death of this child, the whole family, except the master, housemaid, and cook, went away for 8 or 9 days, and the rooms were thoroughly disinfected.

II. Mr. R. June 30 (Sunday), 5 days after the death of No. 1. Consulted me this morning, complaining of soreness of the throat, so slight that he would have taken no notice of it under ordinary circumstances. There was nothing abnormal to be seen about the throat, and the pulse and temperature were normal. I advised him to see me again in the course of the day if he was not better. He came again in the evening, saying that he did not feel so well; that he had been shivering during the afternoon, and that he was afterwards hot. P. 108, T. 101·4°. Throat slightly congested, no patches; tongue coated. Is evidently frightened. Sent him home and to bed. To take the following:

Pulv. Ipecac. gr. 30., s.s.

℞ Tr. Aconit. ♏32, Aq. ad ℥iv.

ʒi every $\frac{1}{4}$-hour four times, then every hour.

July 1st. In bed; not complaining, but trying to convince me there was nothing much the matter with him. The powder made him vomit once. P. 94, T. 99·6°. Slight ulceration in back of pharynx, tonsils and throat slightly congested, no appearance of diphtheritic patches.

6 p.m. Got up after I left him this morning. Has taken the 32 doses of medicine. P. 80, T. 101·6°. Tongue clean and moist. Throat feels more sore, but remains the same in appearance. Has no other feeling of illness whatever.

℞ Liq. Ferri Perchlor. ʒiv, Potass. Chlorat. ʒij.

Glyc. ʒiij, Aq. ad ℥vj.

℥ss. every 2 hours in water.

July 2nd. 11 a.m. P. 88, T. 99·6°. Throat less sore. A

few white specks on the tonsils, the size of a pin's head; some moved away by gargling water, others fixed. Feels perfectly well in other respects.

8 p.m. P. 82, T. 100·4°. Rep. mist.

July 3rd, a.m. P. 80, T. 97·4°. Feels all right. Throat slightly congested, no patches. Is going to his office in a cab.

Mist. three times to-day, twice to-morrow, and then stop.

III. Housemaid, July 6th. Sore throat and malaise, with foul tongue, and pulse 100. Treated by iron and chlorate of potash, and was well again in 4 or 5 days. No patches on throat.

IV. E. R., æt. 3. July 8th. Almost suddenly ill with sore throat, thirst, and general malaise. Tonsils and throat congested, two white patches on former. P. rapid, T. 100·4°. In bed.

℞ Liq. Ferri Perchlor. ʒij, Potass. Chlorat. ʒi.

Glyc. ʒij, Aq. ad ℥ij.

ʒi. every 2 hours in water.

10th. Rep. mist. every 4 hours. Temp. normal yesterday. The spots have disappeared, and the child seems much better in every way, taking her ordinary food, and running about.

August 13th. Diphtheritic patch on left tonsil, size of threepenny-bit. P. 120, T. 102·2°. Was running about in the garden half-an-hour before I saw her, at mid-day.

℞ Liq. Ferri Perchlor. ʒij, Potass. Chlorat. ʒi.

Glyc. ʒij, Aq. ad ℥vj.

℥ss. every 2 hours.

14th. P. 108, T. 100·4°. Seems better. Rep. mist.

15th. T. 98·4°. Tongue patchy. Throat clearing. Was in bed yesterday, but to-day is running about in the nursery. Mist. every 4 hours.

16th. P. 120, T. 103°. Ill again since last night. Right tonsil swollen, and a large patch on it. Some outside swelling of neck. Mist. taken every two hours since last night.

17th. P. 96, T. 98·4°. Thick whitish patch on right tonsil, loosening. Outside swelling lessened. Child much livelier. To stop in bedroom. Medicine every two hours to-day, every four hours to-morrow. Ordinary food to-morrow.

19th. Doing well. Patch peeling off tonsil. P. and T. normal.

20th. Patch off. Rep. mist. t.d.s.

V. W. R., æt. 8. Attacked Aug. 8. Called to him at 11 p.m. P. 132, T. 103·2°. Headache. Throat not complained of till asked about it, when he said he had slight pain on swallowing. Tongue clean, dryish. Small whitish patch on each tonsil.

℞ Potass. Chlorat. ʒi, Liq. Ferri Perchlor. ʒij.

Glyc. ʒij, Acid. Hydrochl. dil. ʒi, Aq. ad ℥vj.

℥ss. every 2 hours.

Aug. 9th. Patches much extended on tonsils, which are swollen. Says his throat feels worse. P. 120, T. 102·6°. *Vesp.*, P. 120, T. 101·4°. Patches greenish, dirty-looking, separating. Has vomited 3 or 4 times. Cont. mist.

10th. P. 92, T. 98·8°. Remains the same. Cont. mist.

11th. P. 96, T. 99·2°. Seems better himself, but speaks more thickly. Outside of neck below angle of jaw much swollen (not so much as last night, the nurse says). There was slight glandular swelling there yesterday morning. Bowels relieved by an enema. Cont. mist.

12th. P. 96, T. 98·8°. Outside swelling less. Throat patches separating. Meat for dinner yesterday. Takes his food much better, also fruit. Cont. mist.

13th. P. 96, T. 98°. Throat clear of patches, leaving irregular depressed surfaces on each tonsil. Outside swelling much less. Mist. every 4 hours. (Another child now ill, No. IV.)

VI. The cook. Seen on Aug. 9th, the day after first seeing No. V. Found her in bed, complaining very much of her throat. Has been out of sorts for a week. Would not have stopped in bed, only "her head ached very much, and she felt so giddy." T. foul. P. 116, T. 101·4°. On the tonsils there are greyish-looking patches.

℞ Tr. Aconit. ♏32, Aq. ad ℥iv.

ʒi. every $\frac{1}{4}$-hour four times, then every hour.

10th. P. 112, T. 102·4°. Perspiring. Feels no better.

℞ Liq. Ferri Perchlor. ʒiv, Potass. Chlorat. ʒij.

Acid. Hydrochl. dil. ʒij, Aq. ad ℥vj.

℥ss. every 2 hours in water.

11th. Eight doses of medicine taken. Feels much better. Right tonsil not so much swollen, but it and the left tonsil have greyish and greenish-grey patches. P. 72, T. 98°. Feels very hungry. To get up and have her ordinary food. Mist. every 4 hours.

12th. Up. Throat quite clean. Feels well, but weak.

Quinine gr. i. thrice daily.

*Remarks.*—Diphtheria, as we know, is a constitutional disease, acute and specific, with local manifestations chiefly referable to the throat and air passages. It is infectious. But the intensity of the infection is variously stated by writers on the subject; some saying that actual contact of a mucous membrane with the discharges from the throat is necessary to convey the disease, whilst others assert that the poison clings tenaciously to houses and rooms for an indefinite period. I am inclined to adopt the first view. I think that the contagiousness of this disease is limited, and that there is but little risk, provided that proper precautions are taken to ensure immunity from contact with septic discharges and with the breath of the patient. In cases that have been put down to the poison clinging to houses and rooms, is it not likely that the cause may have been the continuance in operation of the cause which gave rise to the first case? I think this more than probable.

As to the *causes* of diphtheria, Dr. Wilson, in his *Handbook of Hygiene*, says: "There are good grounds for believing that the disease is sometimes originated by impure water, or defective sewerage." And a writer, some few years ago, in the *British and Foreign Medico-Chirurgical Review*, in reviewing the whole subject, states that "the proofs that it and enteric fever are both dependent on sewage gases, or what escapes from them, are very much the same."

Assuming that the disease is contagious, and that defective sewerage arrangements have something to do with it in many cases, where was the cause of this particular outbreak to be found? I believe that No. I, or rather the previous case which I did not see, was the first case that had occurred in the town for some time; there was, therefore, no epidemic, and enquiry failed to elicit the exposure of any member of the family to infection.

Then the drainage, the drinking water, and the milk supply, naturally occurred to one. The milk vendor supplied many other families, and in no single instance could it be ascertained that there was any specific disease in such families. The water arrangements of the house, so far as one could judge, were not at fault; and the W.C. arrangements were, on enquiry, repeatedly *said* to be in perfect order; but, after much questioning, I elicited this fact, that, at times, there was undoubtedly a very offensive smell from the W.C. Now, taking this fact in connection with the fact that the W.C. door was close to the nursery door, at the end of a longish dark passage without ventilation, and that the master of the house, impressed with the necessity of a thorough and constant current of air through the W.C., kept the *door* as well as the window constantly open—thus ventilating his W.C. through his nursery!—I don't think we have far to go for the cause of his diphtheria, more especially when it is considered that his house was situate in a street notorious for outbreaks of zymotic disease. The fact that the W.C., under such an arrangement, was more frequently ventilated *through the nursery* than through the window, was patent enough to my friend when I explained it; but still "he was not happy." A veritable St. Thomas, he wanted proof positive as to such a state of affairs having anything to do with his diphtheria as a cause. I could only reply that in many cases, where *positive* proof could not be obtained, we might equally rely on *presumptive* proof, and that, to my mind, there was quite sufficient presumptive proof that the defective W.C. arrangements in his house caused this outbreak of diphtheria. (A subsequent examination of the W.C. proved that its condition was about as bad as it could be.)

Let me state briefly the sequence of cases:

| | | | |
|---|---|---|---|
| 1. F. child, æt. 9½ | No medical attendance | | Recovered |
| 2. do. æt. 5½ | Ill June 20th | to 25th | died |
| 3. Master | " " 30th | to July 3rd | Recovered |
| 4. Housemaid | " July 6th | to 10th | " |
| 5. F. child, æt. 3 | " " 8th | to 9th | " |
| (Relapse) | Aug. 13th | to 15th | |
| ( " ) | " 16th | to 20th | |
| 6. M. child, æt. 8 | Ill " 8th | to 13th | " |
| 7. Cook | " " 9th | to 12th | " |

The room in which the child died was thoroughly disinfected, and the whole family, except the master, housemaid, and cook, went away for a week about the 27th or 28th of June. It may be interesting to note that the next two persons attacked were two of the three left in the house; also, that the other members of the family remained free from any signs of the disease until two or three days after their return home. This would seem to point to some common cause for each attack, as also would the curious fact that there was an interval of more than a month (from July 9th to Aug. 13th) during which there was no diphtheria in the house. This was most fortunate, although I am unable to explain it; for the mistress of the house was prematurely confined (8 months) on July 24th, the child only living about 24 hours. She did well, although her convalescence was not so rapid as on previous occasions.

How are we to differentially diagnose diphtheria? I believe in many instances it is exceedingly difficult to do so, and I believe that not unfrequently mild cases of diphtheria occur, which are simply called "sore throat." The series of cases I have here detailed was undoubtedly diphtheritic; but had Nos. 2 and 3 occurred as isolated cases, would any one have called them diphtheria? I doubt it. As occasional aids to diagnosis we have the occurrence of albumen in the urine during an attack, and the more rare occurrence of paralytic symptoms as a sequel. Some physicians, I believe, go so far as to say that albumen in the urine is the one diagnostic point in diphtheria. In case No. 5, I tested the urine for albumen several times, with a negative result; but a week after convalescence had set in, the patient suffered from paralytic symptoms referable to the throat, for which he was put on a four weeks' course of iron and strychnine, and he was well. In Quain's *Dictionary of Medicine* it is stated: "The general symptom never absent is *prostration of strength;* and the local symptom, absent only when the patient dies before there has been time for its formation, is the formation of false membrane." This series of cases will scarcely bear out such positive statements.

As to *treatment* I shall say but little. We have a zymotic disorder, gravely impressing, as a rule, the blood and nervous system, with local manifestations in or about the throat. We must deal with these, and we must avert the tendency to death which is by asthenia or by suffocation. *Aconite,* admirable as it is as a remedy in many inflammations (and its ability to reduce the pulse and temperature is well shown in case 2), is of no use here in the general treatment. The *perchloride of iron* appears to me to be the most valuable remedy we can find to meet the indications in this disorder. But it is no use fiddling with it. It must be given in large doses, frequently and regularly repeated, as we would give it in erysipelas, or it is of no use. Of course it is also of importance, as it is in so many acute diseases, that we see the case early, if we are to be successful. All the cases I have here related were treated in this way, and with the iron I combined chlorate of potash. Frequent feeding with nourishing food was also part of the treatment. I think I have reason to be contented with the treatment adopted, as no case lasted more than six days, and all recovered well except the first case. No local treatment whatever was adopted, and I believe if we see the cases early, and treat them energetically, we shall have no occasion for such treatment. A good *emetic*, if given early, is reported by some to put a stop to the disease—to cut it short. In November, 1881, I published a case in the *Lancet* which would seem to support this view; but it is not safe to draw conclusions from one case. An emetic, if the case is seen early, can do no harm, and I should always be inclined to prescribe it. I tried it in case 2, but with no apparent effect.

*Lactic acid* and *sulphurous acid*, in spray, have been favourably spoken of in the local treatment of the throat symptoms; the former, given early in the disease, with the view of dissolving the false membrane. Of these, as of *tracheotomy* later on, to avert the tendency to death by suffocation, I have nothing to say from personal experience.

In the treatment of any disease, where we feel the ground somewhat uncertain, we are apt to use a number of different remedies, and where recovery ensues it is impossible to apportion the share which such remedies may have had in favouring the recovery of the patient. The bias of a man's mind will often influence him in forming his opinion, and, like the man who foreswore water with his whiskey because he only got drunk when he took them mixed, he is not unlikely to give the credit to the wrong remedies. I have no doubt that it is in this way that so many remedies get lauded in the treatment of disease, only to be afterwards discarded as useless. There is great advantage in our medicinal treatment being as simple as possible; for then, where the result is recovery, we are in a better position to appreciate the value of remedies. The bearing of these remarks will be seen in the cases I have here recorded. The treatment was simple and almost single, and, therefore, so much the more useful to one in thinking over matters afterwards. In these days of scepticism about everything, I fear we are too sceptical about the value of drugs in the treatment of disease.

